# ‘I was ‘only’ seen as a birthing woman:’ the pregnancy, birth and postpartum experiences of women with refugee backgrounds in Norway

**DOI:** 10.1186/s12884-025-07624-x

**Published:** 2025-04-26

**Authors:** Hilde Sjobo Asbjornsen, Fungisai Gwanzura Ottemöller, Eline Skirnisdottir Vik

**Affiliations:** 1 Department of Health Promotion and Development, University of Bergen, Bergen, Norway; 2 Department of Health and Caring Sciences, Western Norway University of Applied Sciences, Bergen, Norway

**Keywords:** Maternal health, Pregnancy, Birth, Postpartum, Refugees, Women, Health inequities, Intersectionality

## Abstract

**Background:**

Norway is recognised for its high-quality maternal healthcare. However, health equity has yet to be achieved. This study explored the experiences of women with refugee backgrounds during pregnancy, birth, and postpartum in Norway. We aimed to understand how the diversity of their backgrounds and current life circumstances, influenced the women’s health, well-being, and interactions with maternal healthcare services. The study focused on the perspectives of women whose needs have not been fully acknowledged in maternal healthcare services both at national and local levels.

**Method:**

Consistent with our Feminist Participatory Action Research approach, we included multicultural doulas, women with lived experience of migration and giving birth in Norway, as co-researchers. We also collaborated with practitioners such as midwives and other relevant actors. We recruited and conducted qualitative interviews with ten women with refugee backgrounds who had given birth in Norway.

**Results:**

Our findings revealed that the women’s intersecting identities were crucial in shaping their maternal healthcare needs during pregnancy, birth and postpartum. The structural inequities faced in maternal healthcare services during pregnancy such as lack of language and communication support, limited access to social support networks, and unfamiliarity with healthcare services were also apparent during birth and postpartum. Adjusting to a new country while raising children compounded these challenges, impacting experiences of pregnancy, birth, and postpartum.

**Conclusion:**

This study highlights the importance of adopting an intersectional approach to maternal healthcare, and not treating race, class, gender and migration experiences in isolation, but taking them into consideration when designing and implementing services. Our results suggest that current policies and services often overlook the specific needs of women with refugee backgrounds. To achieve true health equity in maternal healthcare services, policies should prioritize these women’s unique needs and experiences, ensure that services are adapted and properly funded, and that initiatives guarantee active participation and representation from women with refugee backgrounds.

## Background

Globally, it is estimated that out of the 43,7 million refugees, around 49 per cent are women and girls [1]. In addition, an estimated two million children were born as refugees between 2018 and 2023 [1]. Previous studies show that being a refugee and pregnant can be risk factors for health and wellbeing as there is a tendency for negative pregnancy related outcomes such as maternal death, postpartum depression, and stillbirth [2]. Although in many High-Income Countries (HIC) in Europe the general population receives high quality healthcare, services are often not flexible enough to meet the needs of pregnant refugee women [3, 4]. Providing adequate care for women with refugee backgrounds upon resettlement is important, as adapting to life as a new citizen and as mothers is a major life transition [5–7]. In the current global context, maternal health is now framed more holistically. The shift of discourse is evident in international policies, where every aspect of women’s health including the physical, emotional, psychological, and social is now being emphasised [8]. This is important to acknowledge, as good quality of care received before, during, and after birth increases the likelihood of positive health outcomes and well-being [7]. Nevertheless, data concerning refugee women and maternal health is inconsistent, and they are not represented in the drafting of policies and designing of intervention programmes [9]. In Norway, one out of four children is born to a woman with an immigrant background [10]. In 2022, a total of 51,500 children were born in Norway, and 13,300 of them were born to immigrant women [10]. Norway is a HIC, and offers universal, high-quality, and free maternal healthcare services [11]. However, recent studies indicate that the goal of health equity is not being realized [12–14] and there is a knowledge gap of measures to better facilitate maternal healthcare services to an increasingly ethnically and culturally diverse population [11, 12, 15]. This gap was also highlighted in the Women’s Health Strategy in 2024 [16] and in the 2024–2027 National Health and Cooperation Plan [17]. While important research concerning disparities in maternal healthcare have been conducted in Norway [11, 12, 14, 18–22], to our knowledge, there is no study that focuses on women with refugee backgrounds and their experiences throughout pregnancy, birth and postpartum. Therefore, the aim of this study was to focus on the experiences of these women during pregnancy, birth and postpartum in Norway, focusing on their experiences of the maternal healthcare services. Ultimately, the study aimed to address issues that have been largely overlooked in the design and delivery of maternal healthcare services in Norway.

## Methods

### Study design

This study applied a Feminist Participatory Action Research approach, where the methodological framework of Participatory Action Research was combined with feminist theory [23, 24]. We actively involved women with lived experience of migration/forced migration in our study as co-researchers and collaborated with relevant organizations. Working with co-researchers provided opportunities for us to gain more in-depth insights into their experiences both personally, as they shared their lived experiences of settling and giving birth in Norway, and professionally, in their work as Multicultural Doulas. We also collaborated closely with two maternal healthcare service clinics and the introduction center for refugees throughout the study; this allowed for closer connection between practice and research.

### Study setting and data collection

The study was conducted in a city in Western Norway. The largest groups of immigrants and Norwegians-born to immigrant parents in Western Norway come from Poland, Ukraine, Lithuania, Syria, Somalia, Iraq and Eritrea [23]. The inclusion criteria were women with refugee backgrounds aged 18–45 years who had given birth in Norway. During recruitment, we used interpreters, when possible, and used pictures and simple language when presenting to organisations and women’s groups. We developed an information brochure, together with the co-researchers, translated into eight different languages: English, Swahili, Arabic, Dari, Pashto, Somali, Tigrinya, and Kinyarwanda. We did purposive sampling, and recruitment was conducted in several ways: through the co-researchers’ networks, by local actors such as midwives and people working at the introduction center, and by presenting our study at language classes and at women’s groups. This created a snowball effect where women who were interested in participating in the study contacted us. We collected data from September 2023 until April 2024. Ten women from Eritrea, Syria, Lebanon, Kuwait, Liberia, Congo and Somalia were recruited to the study. We developed the semi-structured interview guide in collaboration with the co-researchers to ensure that the questions were appropriate and captured elements around the issues of being a refugee and maternal health experiences in Norway. The women chose the time and place for the interviews. All the interviews were conducted by the first author (HSA) and the co-researchers were present at four of the interviews, at the request of the participants they had recruited; the intention was to create a more relaxed and trusting atmosphere. A narrative approach was used during the interviews encouraging the women to tell their stories and using the interview guide as a prompt. In two of the interviews, the co-researchers also interpreted. The co-researchers received briefing and training from two of the authors (HSA and FGO), but two of the co-researchers were already well-versed with interview techniques and ethical considerations as they are employed by organisations that work with forced migrants. We provided an interpreter where required, and a female interpreter when possible. For three of the interviews, we had to use an interpreter over the phone and one of those interviews had to be stopped due to poor interpretation.

### Analysis

Data was analyzed using reflexive thematic analysis as this method can be used together with different theoretical frameworks [24], and thus we could apply an intersectional lens during data analysis. The first step in reflexive thematic analysis is familiarization of data. We transcribed all the interviews, took notes and added comments to the text. The transcribed interviews were then uploaded into NVivo software program. The next step involved generating codes where we coded the data inductively and developed themes. An overview of the themes was first presented to the co-researchers, and we reflected and further refined them together and discussed how to best present the findings: (see Table 1). This was an iterative process as we went back and forth between the steps in the data analysis and the data. We presented the initial themes and sub-themes to local actors such as midwives, staff working at the introduction centre and a local women’s organization for feedback and as part of our participatory action research approach. As a result, we made an overall thematic table: (see Table 2).

**Table 1:** Example of the analytical process

Quote	Code	Sub-theme	Theme	Relation to theoretical framework	Reflections	Notes from the co-researcher’s reflections
They order a translator, but if you stay in the hospital, let’s say for 4–5 days… I was there for 3 days. They would come and ask me things, but I did not understand them.	1. Did not get an interpreter after giving birth 2. Missing information and difficulties in expressing needs	Language barrier in hospital setting	Lack of language support and communication during birth and postpartum	Structural inequities in maternal healthcare	We need to look at the broader picture in her story. She does not have an education, and did not know how to read or write. She has also previously experienced a stillbirth and has not been used to giving birth in a hospital setting. Many factors interlink and makes it more complex.	A support person before birth, during birth and the time after birth is very important for many of the women with refugee backgrounds. Support before and after birth is very crucial.

**Table 2:** Thematic table

Global Theme	Theme	Sub-theme	Description	Relation to Theoretical Framework
Invisible Identities in a Universal Maternal Healthcare System	The Role of Intersecting Identities in Shaping Maternal Healthcare Needs.	Diverse backgrounds and life Situations. Race, class, gender, immigration experiences.	Impact their access and utilization of healthcare services and overall wellbeing.	Integrational categories are not captured in practice. Policy gaps in viewing women as one category.
Consequences of Structural Inequities in Maternal Healthcare Services	Experiences of Structural Inequities in Maternal Healthcare Services	Lack of language and communication support.	Barriers due to language differences and inadequate translation services. Created fears and misconceptions.	Structural intersectionality: Support systems are not capturing different race, class and gender experiences and the intersection between them, hence needs are not being met. Current practices fail to provide adequate support.
		Limited access to social Support networks.	Emotional strain, lack of community support and isolation affect maternal health.	
		Challenges of being unfamiliar with healthcare services.	Difficulties in navigating the healthcare system as the system is not adapted to individual needs.	
Policy and Practice Inequities in Maternal Care and Support Systems	Adaptation and its Influence on Pregnancy, Birth and Postpartum Experiences	Lack of adaptation support. Lack of tailored and adequate services during settlement in Norway.	Challenges faced by the women in adapting to a new system and managing new roles in a new country and at the same time experiencing pregnancy, birth and caring for children.	Ineffective implementation of existing policies in capturing the needs of women with different life situations and backgrounds.

### Positionality

The research team consisted of women coming from different backgrounds, countries, and life situations. The three co-researchers had all given birth in Norway, and had come from Tanzania, Somalia and Turkey, as young women with their husbands but without their extended families. Two of them came to Norway as refugees, while one came as a student. All of them work as Multicultural Doulas for a local women’s organization. The other team members are employed at Universities; HSA is a white, middle-class woman and a mother, born in Norway. She has experience working on issues concerning women’s health both globally and nationally. FGO is a black southern African woman who immigrated to Norway as a student and now teaches health promotion and conducts research on migration at the same University as HAS. She has experience working as doula in Norway, but not with women with refugee backgrounds. She is also a mother and has given birth in Norway. ESV is a midwife with a Norwegian and Icelandic background. She lectures and conducts research in midwifery related issues including migrant health at a different University, and she has also given birth in Norway. The diversity within our team has been a significant strength, as our varied racial, nationality, and class backgrounds and different life experiences have brought unique perspectives and insights to the topic.

### Ethics

In the implementation phase of the study, the representation of women with refugee backgrounds, highlighting the diversity of experiences within a ‘group’ of women, and ‘giving voice’ were central issues. Using a feminist PAR framework does not automatically ensure ethical practice [25]. However, as Yuval-Davis [26] argues, the research process and the level of involvement are crucial for its implementation As a research team we were aware of how women from forced migrant backgrounds are not represented in policies and practices concerning women’s health in Norway and of the tendency to classify them as ‘one group’ and categorize them as vulnerable. For us, the women in this study were not vulnerable, but rather in vulnerable life situations. Communicating this distinction has been crucial when presenting the study and its findings to broader audiences. Collaboration with practitioners working on maternal health issues and establishing a diverse research team has been important, as it has brought a more equitable approach to the project. Engaging directly with women who have experience with forced migration and pregnancy in Norway was needed to make sure that the study remained relevant and focused. During the data collection phase, we introduced the project in the participants’ preferred language, clarified any expectations or questions, and were mindful of how we asked questions. We also took care when using interpreters, ensuring as far as possible that they were women, and informing them about the interview process and topic beforehand. Informed consent was given by all the participants, both orally and in writing, before the interviews were conducted. In the data analysis stage, we worked together on the analysis with the co-researchers. The presentation of findings was also discussed and done together as a team, keeping in mind representation and issue of ‘giving voice.’ Confidentiality has been key, ensuring that data was coded anonymously to prevent participant identification [27]. Data was stored and analyzed in the University’s secure SAFE data repository, with personal data stored separately from the transcripts in encrypted form with restricted access. This project was also approved by the local municipality and the management of personal data was approved by the Norwegian Agency for Shared Services in Education and Research.

### Theoretical framework

Our study applied intersectionality as a critical lens to interpret and understand the participants’ experiences during pregnancy, birth and postpartum. Collins [27], emphasizes that while the interpretation and application of intersectionality varies among scholars and in practice, it generally highlights how race, class, gender, sexuality, nation, ability, and age are not separate isolated categories, but interconnected forces that collectively shape complex social inequalities. When Kimberlé Crenshaw applied the term in 1989, she showed how multiple forms of discrimination, power, and privilege intersect when shaping the employment situation of Black women in the United States [28]. Crenshaw [28, 29] further pointed out that political and structural intersectionality helps to question existing power structures, dominant discourses, and to showcase the barriers faced by those with intersecting identities, such as race and gender. In the field of health, intersectionality has been applied to better understand the complexity of health inequities [30]. Within women’s health, scholars have explored how mainstream health policies disregard issues like the differences between women, and do not address underlying causes of health inequities, such as discrimination and subordination [31]. Applying intersectionality as an analytical tool, enables us to gain insight into some of the issues faced by women with refugee backgrounds, that are currently not adequately addressed in dominant frameworks or healthcare practices in Norway.

## Results

The findings below illustrate how the women’s experiences of being refugees and their backgrounds and life situations impacted their pregnancy, birth and postpartum experiences, particularly in relation to their access to and use of maternal healthcare services. The cross-cutting issues of language, social support, and unfamiliarity with healthcare services were evident throughout pregnancy, birth and postpartum.

### The role of intersecting identities in shaping maternal healthcare needs

The women came from different countries and had diverse migration experiences, life situations, and backgrounds which impacted their access and utilization of maternal healthcare services. Most were either pregnant upon arrival in Norway or within the first year. Some had formal education, while others were illiterate. Some had travelled with families, others arrived alone, or with their children. Many had prior pregnancy experiences, and a few had experienced stillbirths. Upon arrival in Norway, the women were required to participate in the Introduction Program for Refugees. Being pregnant during the introduction program and managing multiple roles in the household such as being the main caretaker of the children often resulted in the women having to take sick leave or to quit the course altogether. As one mother of seven children explained:

*I was three months pregnant. I was so tired and exhausted. I just wanted to sleep. I did not manage to have control over my body. I was so tired. That is why I needed sick leave from the Introduction Course. It was just so heavy. Getting the children to school*,* getting myself ready*,* rushing home after classes*,* make dinner…So it was hard. (Participant 5).*

It was crucial for the women who did not have any formal education to attend the courses during pregnancy despite lack of sleep and the double burden of household chores. They shared that although learning a new language was challenging, it is a necessity for managing daily life in Norway:

*You are deaf without language. The first thing I tell women when I meet them here at the Women’s Organisation is*,* ‘do not get pregnant before you learn the language.’ When I look back at the time when the kids were young*,* I find it very challenging*,* like wow*,* it has not been easy to live in a new country. (Participant 8).*

In contrast, the findings indicated that the women who had higher education before coming to Norway tended to become pregnant later, usually after a few years. They initially continued their education, and learnt Norwegian through education, job training and social networks. As most of the women did not have networks upon arriving in Norway, they expressed that being away from family during pregnancy added extra emotional strain:

*Many get depressed and are very lonely. You do change a lot during a pregnancy. Maybe you have a lot of children at home also. It is tough. You need someone who can be with you and support you during pregnancy. Even though you have a husband*,* you do not necessarily have your family around you. I have no family here. (Participant 8).*

For the ones who had stayed longer in Norway before getting pregnant, the dependency on social networks was important during pregnancy. However, despite having a social network, being pregnant and not having family around was still an emotional strain.

### Experiences of structural inequities in maternal healthcare services

The women’s stories indicate that the maternal healthcare services did not consider the women’s different experiences and identities, leading to misconceptions, insecurities and trust issues when encountering the maternal healthcare system. Many of the women shared that their first point of contact during pregnancy was with a general practitioner, as they were unaware of the maternal healthcare clinics and the services they provide. Once referred to these clinics, the women generally expressed that the care provided by the midwives was good, and they valued being closely followed up during pregnancy. However, on sharing their experiences of the visits, they highlighted various barriers such as access and lack of interpreters during appointments:

*My first challenge was that I did not have the language skills. When I had an appointment with a midwife*,* I could sometimes not go to the appointment. I had to call someone I knew to see if they could join me*,* or someone who knew English who could join. Going to the midwife was also very difficult. We do not know the area*,* so we were very dependent on a contact person who could drive us back and forth. (Participant 2).*

The women expressed that not being able to communicate or receive information in their native language was difficult. Most of the women also shared that the information and support they sought during their pregnancies, was provided through informal networks, such as social media or people from their countries of origin.

*If you do not have the language*,* you are unable to search online for information*,* travel to the healthcare facilities*,* call the midwife to get easier access to services. The knowledge the women have about the system is often not because they asked health personnel*,* but because friends told them. I tell them that they need to ask the midwife because that information is often better. (Participant 7).*

Inconsistency in the use of interpreters was also an issue highlighted by the women, with some saying that they had an interpreter for some of the appointments, while others had none. However, they shared that they rarely had in person interpretation, it was mostly conducted remotely over the phone. Some of the women’s concerns when preparing for birth indicate inadequate support from the maternal healthcare services:

*The fear… I started to dread giving birth. It has given me great worries. I thought about the consequences if something were to happen to me during birth. I also worried about who would take care of my children when I was giving birth. What happens after birth? So that time during pregnancy was mixed with stress*,* sadness*,* and worries. I had no one to talk to about these feelings*. *(Participant 5).*

For some of the women, meeting a support person before birth was crucial and helped them to feel more secure during pregnancy and preparing for birth at the hospital. One woman met a person who worked as a Multicultural Doula at an Eid party and was eager to get this support. She was not aware that this support was free of charge and available but felt very relieved when she managed to get it:

*I felt safe with this support. When the Multicultural Doula came with me to appointments*,* I then got the support I needed…I have told all my friends who are pregnant about this support*,* as they do not know about it. I have told them that they need to ask for it. (Participant 5).*

When it came to giving birth, the women experienced similar challenges with maternal healthcare services as during pregnancy. Although the women came from countries where the maternal healthcare services might not have been as comprehensive as those in Norway, they had the security of family support during birth. Many of the women became pregnant within a year of arriving in Norway, they had not been in a Norwegian hospital before and felt insecure about the system. They recounted their previous birthing experiences and shared how they had missed having someone to support them:

*I will never forget the day I was going to the hospital to give birth. We have as a tradition that your mother or mother in-law*,* sister or other relatives will come with you. I had to go to the hospital by myself as my husband had to stay at home with the children. (Participant 2).*

It was painful for them to look back on those experiences:

*When I think back at the time when I was going to give birth after arriving in Norway*,* I get back some of the feelings like the pain you know. Every time I meet someone who is pregnant*,* it reminds me of the painful experience I had. (Participant 2).*

As one woman shared, having had difficult births experiences when she was very young in her home country, such as stillbirth, the support of someone she could trust during childbirth helped her feel more comfortable and safer:

*One day I met a lady*,* she was from the same country as me*,* and she said*,* ‘does your mother live here?’ I said ‘no*,* I live alone’. She told me that if I needed help*,* I should call her. She was with me when I was about to give birth. I didn’t want to have a C-section. Only if she was there. (Participant 9).*

Language was a significant issue for most of the women during birth as many of them did not speak Norwegian or English. Having an interpreter was important to help them express their needs, ask questions, and understand the information provided. However, what came through in many of the women’s stories was that they did not get this service, or it was inconsistent. This resulted in the women feeling nervous and anxious:

*I was in the hospital for three days. They would come in and ask me things*,* but I didn’t understand anything. I got scared [woman shows with hand movements that her heart was beating] when the doctor came into the room. (Participant 9).*

The lack of communication in their native language resulted in many of the women not knowing what was happening:

*The day after giving birth*,* I thought there was something wrong with the baby since I had to go to the doctor for a check-up. I did not know what it was. No one was talking to me*,* so I did not know what was going on. (Participant 10).*

While the birth experiences in Norway highlighted significant challenges related to social support and language, these challenges continued to influence the women’s postpartum care experiences. Home visits from a midwife are an important aspect of postpartum care in Norway, and yet many of the women were not aware of this service. Most of them were pleased with the home visits after giving birth. However, many did not know beforehand what the home visits entailed. They wished that this information had been communicated to them prior to giving birth especially as some of them were misinformed about the visits:

*People told me beforehand something that was wrong. They said that those who come on the home visits will try to check what you have bought for the baby*,* what you have in your closet*,* and things like that… However*,* they did not come to check the cupboards and what I had bought. They were nice and respectful. (Participant 3).*

In addition, the lack of an interpreter during the visits, made it difficult for the women to express their needs and concerns:

*I had visitors for half an hour*,* maybe an hour. There was no interpreter*,* so it was not easy to talk about these (difficult) feelings that I experienced. It was not. (Participant 2).*

In Norway, the maternal healthcare services offer postpartum groups as a support to mothers. Some of the women were offered the opportunity to participate in postpartum groups, but as they were only conducted in Norwegian or English, many of the women could not participate. Moreover, a few women also expressed that the postpartum groups were not appropriate for them, as they felt the sessions were time consuming considering their busy lives and involved too many questions.

### Adaptation and its influence on pregnancy, birth and postpartum experiences

Adapting to a new country and new systems is a challenging process for most immigrants, and being pregnant, giving birth and going through postpartum further exacerbates this process.

The women shared that it was challenging to adapt to a new country, climate, adjust to new roles within the household, attend language courses, and simultaneously manage their children’s transitions into kindergarten or school. One of the women illustrated this by explaining the differences in her pregnancy experiences in Norway and her home country:

*The last pregnancy was difficult because of the language*,* new country…I can say it another way*,* all the five previous pregnancies on one side*,* and the last one on the other side*. *(Participant 2).*

Furthermore, navigating parenthood with limited extended family support was very stressful, and some of the women said they left the hospital earlier than recommended because they had to return home and take care of their children. In addition, they also had to face new roles and expectations outside the home because of the introduction program. For some, this resulted in quitting language courses and/or education after having children. One of the women who was currently on maternity leave was concerned about going back to school:

*The best thing about our country is that we are always with our children. Now it is very difficult for me*,* and I just think about the fact that he will soon go to kindergarten when I finish my leave.*

She expressed a wish to stay at home longer, but did not feel she had a choice because of the rules that regulate the monthly payments she received:

*It is a lot of pressure. The people at the Introduction Center for Refugees are only saying that I need to start classes again and the baby needs to go to kindergarten. (Participant 5).*

The variety of concerns many of the women were facing had an impact on their health and well-being:

*I feel quite a mess. I have backache*,* many children at home*,* and soon I must start work practice. I miss my family. My father is ill. I haven’t seen them in 13 years. Things are not quite right…I have 6 children you know*,* and a lot of absences from the introduction program. It causes a lot of problems for me. (Participant 2).*

Despite wanting to adapt, many felt overwhelmed. When asked if they had expressed their needs to healthcare personnel or staff at the introduction center, some of the women explained that they wanted to manage on their own. Limited information about the supportive role of the social services led to some women expressing fear of sharing their worries with the healthcare workers:

*To be 100% honest*,* it is scary to contact the healthcare services. You know*,* they have an obligation to report to childcare services. Despite that you have not done anything to your child*,* but if you are saying that you are tired*,* or need some kind of support*,* that also means that someone else needs to take care of your child. And then the childcare services will enter. (Participant 1).*

Moreover, many of the women expressed a desire to support other women in similar situations as they reflected on their own past experiences:

*When I see someone who is pregnant here at the language course*,* I care a lot. I’m starting to think about them. How are they going to cope? The memories always come back to the experience I had*,* and I start to put myself in their situation and get worried about them. (Participant 2).*

This reflection not only highlights a concern for others’ wellbeing, but also the ongoing challenges many of the women in our study continued to face, from language barriers to feeling isolated, as they navigated their path to settling in Norway throughout the pregnancy, birth, and postpartum period.

## Discussion

The findings highlight how having a refugee background, combined with diverse socioeconomic backgrounds and caregiving responsibilities, shaped women’s access to and engagement with maternal healthcare services in Norway. Structural barriers such as limited access to interpretation, limited social networks, and unfamiliarity with healthcare systems, resulted in emotional strain and inconsistent use of services. The requirement to continue to participate in the Introduction Program for refugees during pregnancy and after birth further illustrates the intersecting demands these women navigated. These findings emphasize how overlapping social positions influence maternal health experiences within a universal healthcare system.

### Invisible identities in a universal maternal healthcare system

As seen in Crenshaw’s work [28, 29], race and gender discrimination do not exist in isolation but interact, resulting in experiences that are not captured when these categories are considered separately. Applying an intersectional lens to our findings enables us to gain insight into the complexities of these women’s realities and how their pregnancy, birth and postpartum experiences are influenced by their socio-economic backgrounds, forced migration/refugee status, and current life situations. Thus far, the maternal healthcare services in Norway have made limited efforts to address these complexities and, as indicated in our findings, this impacts women’s well-being and their utilization of services. Previous studies [5, 32, 33] have shown that it is difficult to provide support tailored to women with refugee backgrounds’ needs when pregnant, if their backgrounds and current life situations are not taken into account. Moreover, while support is crucial, studies have also identified the risk of adverse pregnancy outcomes when a thorough obstetric history and medical records are lacking [34, 35]. In our study, when healthcare personnel did not consider the women’s backgrounds and current life situations, it often resulted in inadequate use of antenatal services, stressful and negative birth experiences, and an overall reduced sense of well-being in the postpartum period. Thus, to effectively address maternal healthcare needs, it is essential to consider how women’s diverse backgrounds and living situations intersect, ensuring that healthcare systems are responsive to these factors.

### Consequences of structural inequities in maternal healthcare services

Providing women with refugee backgrounds the same level of healthcare as general populations, during pregnancy, birth and postpartum often leads to inconsistencies in service use as these do not adequately address their specific needs [32]. In our study, the issues related to language, social support, and unfamiliarity with maternal healthcare services highlighted these structural inequities. In Norway, all immigrants who cannot speak Norwegian have the right to an interpreter when accessing health and social welfare services [36]. However, as seen in previous studies, despite having the right to an interpreter, this support is often inconsistent and insufficient to address women’s needs and inform them about medical procedures [37, 38]. Additionally, studies have revealed that interpretation services need to be professional and aligned with other services aimed at women with refugee backgrounds [32, 39, 40]. This resonates with our findings, as the women expressed not only a need for interpretation but also for someone to explain the procedures, norms and cultural customs regarding having a baby in the Norwegian context. The lack of interpretation during antenatal care, births, and postpartum care, along with the unavailability of postnatal classes in languages other than Norwegian and English, prevented many of the women from expressing their needs and receiving adequate information. This resulted in some women being reluctant to participate in maternal healthcare initiatives. Thus, this lack of language and communication support during pregnancy, birth, and postpartum created obstacles in accessing and receiving adequate maternal healthcare.

Crenshaw [29], writes that when support systems are designed based solely on the needs of women from specific racial and class backgrounds, they fail to address the unique obstacles faced by women whose experiences differ due to race and class. While initiatives such as the Multicultural Doula program were helpful to the women who received this support during birth, they also expressed that it would have been helpful to have consistent doula support throughout. Studies from Sweden [41, 42] show how community-based bilingual doulas assisted women in addressing their diverse needs during childbirth and supported the midwives in their work. Schytt et al.‘s [43] randomised controlled trial study indicated that community-based bilingual doulas “neither improved migrant women’s overall ratings of care for labour and birth, nor their emotional well-being postpartum” (p.16). On the other hand, our findings show that while birth support is important, the needs of women extend beyond the delivery room. Support before and after birth, such as childcare arrangements, the ability to communicate their needs, and transportation to healthcare facilities, were equally crucial. Additionally, we found that navigating parenthood with limited support while also adapting to the Norwegian context impacted the women’s well-being. These experiences are often overlooked in the existing social welfare and healthcare services. Similar findings from other studies, show that social support systems need to consider the different needs of the women throughout the stages of pregnancy, birth, and postpartum [6]. This highlights a critical gap where existing maternal healthcare initiatives and social welfare policies do not sufficiently account for the intersecting factors of race, class, and gender. Healthcare services should adjust their priorities to better reflect the diverse experiences and backgrounds of the women, taking into consideration the interlinkages of, race, gender, and class to ensure that support networks are inclusive and effective in meeting the comprehensive needs of the women during and after pregnancy. The lack of culturally appropriate services has been shown in previous studies in Norway among women with migrant backgrounds [11, 19], and in international studies concerning women with refugee backgrounds [6, 44]. This also emerged as a recurring theme in our study. Lack of familiarity not only created barriers to accessing services, but also misconceptions and fear among women around social welfare systems such as the child protection services. This highlights that the issue is not necessarily a lack of willingness to accept support or that the women do not need support, but rather that the support offered is not rooted in an equitable and inclusive framework. Thus, the lack of familiarity with healthcare services during pregnancy, birth and postpartum can exacerbate healthcare disparities.

### Policy and practice inequities in maternal care and support systems

The Norwegian healthcare system and policies are designed around a universal approach. This does not always consider the diverse needs of women with different cultural and socio-economic backgrounds [15]. According to Crenshaw’s work on structural intersectionality [29], when reforms overlook the distinct position of women of color in the economic, social, and political realms, they are less likely to have their needs met compared to women who benefit from racial privilege. A study conducted in Iceland highlights how discourses of mothering and parenting are often centered on the understanding that the ideals of white, middle-class women are normative [45]. As seen in our study, providing women with refugee backgrounds the same healthcare during pregnancy, birth and postpartum as the general population often led to inconsistencies in service use as the services did not adequately address their specific needs. This reflects the gaps outlined in the 2024 Women’s Health Strategy [16] and in the 2024–2027 National Health and Cooperation Plan [17], highlighting the need for more knowledge about these women and their families’ experiences during pregnancy, birth and postpartum. In addition, in Norway it is obligatory for people who come as refugees to follow the Introduction Program to learn the Norwegian language, gain insight into the Norwegian context, and prepare for work or education [46]. While this program can be an asset for adapting to life in a new country, for some of our participants, it became a struggle to attend courses while adapting to a new culture, caring for children, and being pregnant. This often made it difficult for them to continue language classes and to follow the Introduction Program. The struggle of taking care of children and lack of extended family support has also been highlighted in a research report [47] where as a result people quit and do not proceed with education or job qualifying activities such as internships. Hence, the current policies and practices are ineffective in addressing the diverse needs of women with different backgrounds and living situations. By not considering the needs of women with refugee backgrounds, maternal healthcare services will continue to perpetuate inequitable healthcare.

### Limitations

While this study aimed to cover the women’s pregnancy, birth, and postpartum experiences, a more in-depth focus on a single dimension could have allowed for greater detail in our findings. Moreover, the relatively small group of 10 participants, which, although diverse in terms of nationalities, backgrounds and experiences, may not fully capture the wider forced migrant population. Despite having a small participant group, after a few interviews, our data indicated that the women were facing many of the same barriers. The recruitment process, while thorough and mindful of language barriers, cultural differences, and building trust, proved time-consuming. Furthermore, the use of interpreters during some of the interviews presented some difficulties. Conducting interviews over the phone occasionally interrupted the flow of the conversations and posed challenges in ensuring accurate translation of questions and responses. Additionally, the positionality of the first author, who conducted the interviews could have influenced both the information shared by participants and the level of trust established. To address this, we made efforts to collaborate with our co-researchers and women’s groups and work in solidarity with participants, acknowledging these dynamics as an essential part of the research process.

## Conclusion

This study highlighted the importance of understanding and addressing women’s interconnected identities in shaping maternal health needs, as well as how structural inequalities intersect, creating multiple inequities during pregnancy, birth, and postpartum. Their experiences with healthcare services cannot be viewed in isolation; they must be understood within this complex context to fully grasp the factors contributing to inequities in maternal health in Norway. Without accounting for the diversity of women, the healthcare system fails to provide adequate support, leading to negative outcomes for the women’s health and wellbeing. To make progress in addressing inequities in women’s health in Norway, there needs to be a change in policy and within the practice field, specifically within the maternal healthcare services, and broadly across the social and welfare system. This shift requires a more inclusive and personalized approach, that addresses the specific needs of women with refugee backgrounds, throughout pregnancy, birth and postpartum and a shift away from merely seeing them as ‘birthing women.’

## Data Availability

The data generated and analysed during the current study are not publicly available due to protection of individual participants’ privacy and confidentiality but are available from the corresponding author on reasonable request.

## Abbreviations

HIC: High Income Country
MDGs: Millennium Development Goals
PAR: Participatory Action Research
SDGs: Sustainable Development Goals
UNDP: United Nations Development Programme
UNHCR: United Nations High Commissioner for Refugees
UNICEF: United Nations International Children’s Emergency Fund
WHO: World Health Organization
